# Pattern and Predictors of Medication Dosing Errors in Chronic Kidney Disease Patients in Pakistan: A Single Center Retrospective Analysis

**DOI:** 10.1371/journal.pone.0158677

**Published:** 2016-07-01

**Authors:** Ahsan Saleem, Imran Masood

**Affiliations:** Department of Pharmacy, The Islamia University of Bahawalpur, Bahawalpur, Punjab, Pakistan; Emory University, UNITED STATES

## Abstract

**Background:**

Chronic kidney disease (CKD) alters the pharmacokinetic and pharmacodynamic response of various drugs and increases the risk of toxicity. The data regarding the pattern and predictors of medication dosing errors is scare from the developing countries. Therefore, the present study was conducted to assess the pattern and predictors of medication dosing errors in CKD patients in a tertiary care setting in Pakistan.

**Methods:**

A retrospective study design was employed and medical charts of all those CKD patients who had an eGFR ≤60ml/min/1.73m^2^, hospitalization ≥24 hours, and admitted in the nephrology unit during January 2013 to December 2014 were assessed. Descriptive statistics and the logistic regression analysis were done using IBM SPSS version 20.

**Results:**

In total, 205 medical charts were assessed. The mean age of patients was 38.64 (±16.82) years. Overall, 1534 drugs were prescribed to CKD patients, of which, nearly 34.0% drugs required dose adjustment. Among those drugs, only 41.8% were properly adjusted, and the remaining 58.2% were unadjusted. The logistic regression analysis revealed that the medication dosing errors were significantly associated with the CKD stages, i.e. stage 4 (OR 0.054; 95% CI [0.017–0.177]; p <0.001) and stage 5 (OR 0.098; 95% CI [0.040–0.241]; p <0.001), the number of prescribed medicines ≥ 5 (OR 0.306; 95% CI [0.133–0.704]; p 0.005), and the presence of a comorbidity (OR 0.455; 95% CI [0.226–0.916]; p 0.027) such as the hypertension (OR 0.453; 95% CI [0.231–0.887]; p 0.021).

**Conclusions:**

It is concluded that more than half drugs prescribed to CKD patients requiring dose adjustment were unadjusted. The predictors of medication dosing errors were the severe-to-end stages of chronic kidney disease, the presence of a comorbidity such as hypertension, and a higher number of prescribed medicines. Therefore, attention should be paid to these risk factors.

## Introduction

Chronic Kidney Disease (CKD) is a serious public health concern that affects nearly 10 to 15% of the adult population worldwide [1–4]. CKD is defined as a reduced GFR (glomerular filtration rate) for ≥ 3 months, that may or may not be coupled with a kidney damage, and classified into several stages on the basis of GFR [5]. CKD seriously alters the pharmacokinetic and pharmacodynamic response of various drugs that are mainly excreted from the body through renal route, either in an intact form or in a metabolite form, and increases the risks of toxicity by delaying drug excretion [2, 6].

Studies report that the doses of renally excreted drugs are not being adjusted properly in the hospitalized CKD patients all over the world [7–12]. The percentage of prescriptions having inappropriately adjusted drugs may range from 25–77% during the hospitalization of CKD patients [13–16]. Additionally, it has been reported that nearly 63% of these prescriptions potentially have adverse consequences, and nearly 3% potentially have fatal or severe consequences [17]. However, despite these facts and figures and the seriousness of chronic kidney disease, the renal functioning is still being assessed using serum creatinine values alone in usual clinical practices, especially in the developing countries, which indicates a higher risk of medication dosing errors in hospitalized CKD patients [7, 9].

To address the importance of the issue, a lot of work has been done worldwide to assess the pattern of medication dosing errors, however, very less attention has been paid to assess the predictors of medication dosing errors in CKD patients, especially in the developing countries [8, 9, 11, 12, 18]. From the developed world, a study conducted in the Netherlands has reported that the risk of unadjusted dose prescribing was higher in CKD patients with a creatinine clearance less than 35mL/min/1.73m^2^ [14]. Another study conducted in the United States of America has reported that the inappropriate prescribing in CKD patients was associated with the older age above 85 years, obesity, and the presence of a comorbidity [19]. Likewise, a study conducted in Australia has also reported a higher risk of inappropriate prescribing in CKD patients with an older age, diabetes, and higher number of prescribed medicines [20].

Nevertheless, a few studies were also conducted in the developing countries, but unfortunately they did not yield significant outcomes. For instance, a study conducted in Palestine during 2007 reported that the age, gender, and the stages of chronic kidney disease were not associated with medication dosing errors in CKD patients [9]. Similarly, another study conducted in Ethiopia has endorsed the Palestinian study that gender and the stages of chronic kidney disease are not associated, but the age above 60 years is associated with medication dosing errors in CKD patients [21]. However, to get a clearer insight, more evidence based data is required from the developing countries such as Pakistan, where in the best of our knowledge no such studies are conducted. Therefore, the present study was conducted to assess the pattern and predictors of medication dosing errors in hospitalized CKD patients in Pakistan.

## Material and Methods

### Study Setting

The present study was conducted at the nephrology unit of a 1400-beds, fully equipped, tertiary care teaching hospital named Bahawal Victoria Hospital (BVH) that is situated in Bahawalpur, Punjab, Pakistan. The hospital caters a large population living in the Southern Punjab region. In contrast to other units of BVH, the nephrology unit is comparatively new, and consists only of 15-beds. Patients at the nephrology unit of BVH are being served by trained nephrologists and nephrology residents.

### Ethical Considerations

This study was approved by the Pharmacy Research Ethics Committee of The Islamia University of Bahawalpur, Pakistan (Reference: 21-2015/PREC). Informed consent could not be obtained from the patients (or next of kin/caregiver in the case of children) for their clinical records to be used in this study. However, permissions were obtained from the hospital administration to access and use the data. Moreover, to ensure the patients’ privacy, the data was anonymized and de-identified prior to analysis.

### Study Design and Sampling Procedure

A retrospective study design was employed and medical charts of all those chronic kidney disease patients, who were admitted in the nephrology unit of BVH during January 2013 to December 2014, for a minimum of 24 hours, with a confirmed CKD diagnosis, and had serum creatinine values mentioned in their medical charts, were included. The stages of chronic kidney disease were not mentioned in the majority of medical charts, therefore, the glomerular filtration rate (GFR) of patients was measured using the Modification of Diet in Renal Disease (MDRD) equation, and only those charts were included that had an eGFR less than 60ml/min/1.73m^2^. Which means, patients included in the study had stages of chronic kidney disease as follows: stage 3 (CrCl 30–59 ml/min), stage 4 (CrCl 15–29 ml/min), and stage 5 (CrCl<15 ml/min). All those medical charts not meeting the abovementioned criteria were excluded. A special data collection form was designed and used to collect the required data (S1 Table).

### Measurement of GFR

In usual clinical practice, creatinine clearance is measured using Cockcroft and Gault’s equation [22]. However, in the present study, the authors were unable to calculate the creatinine clearance values using Cockcroft and Gault’s equation due to lack of data regarding patients’ weight. Therefore, an alternative equation, the Modification of Diet in Renal Disease (MDRD) was used to measure the glomerular filtration rate (GFR). The GFR was estimated by the three-variable version of the MDRD-formula: eGFR (ml/min./1.73m2) = 175 x (serum creatinine (μmol/l)/88.4)– 1.154 x (age in years)– 0.203 (x 0.74 if female) [23].

### Dosing Guidelines and the Assessment of Medication Dosing Errors

Due to the unavailability of any single national drug dosing guideline for CKD patients in Pakistan, four reputed references such as the British National Formulary (BNF-58) [24], the Drug Prescribing in Renal Failure-2007 [25], the Drug Prescribing in Renal Failure: Dosing Guidelines for Adult [26], and the Drug Dosing in Elderly Patients with Chronic Kidney Disease guidelines by Lassiter et al [27], were used. The dose adjustment guidelines were adopted from these references and tabulated in consultation with the nephrologists for all those drugs that were classified as the most commonly prescribed drugs in the unit, during a pilot study (Table 1).

**Table 1:** Dose adjustment guidelines for chronic kidney disease patients.

			Dose Adjustment in CKD		
Drugs	Reference	Usual Doses	GFR > 50 ml/min	GFR 10–50 ml/min	GFR < 10 ml/min
Amikacin	[26, 27]	7.5 mg/kg q12 h	60%–90% q12 h or 100% q12–24 h	30%–70% q12–18 h or 100% q24–48 h	20%–30% q24–48 h or 100% q48–72 h
Amiloride	[26]	5 mg/d	100%	50%	Avoid
Amoxicillin	[26, 27]	250–500 mg q8 h	100% or q6 h	100% or q6-12 h	75–50% or q12-16 h
Aspirin	[24, 26]	81–325 mg/d	100% q4 h	100% q4-6 h	Avoid
Atenolol	[26, 27]	5–100 mg/d	100%	50–75%	25–50%
Azathioprine	[24, 26]	2–2.5 mg/kg/d	100%	100%	75%
Bisprolol	[25]	10 mg/d	100%	75%	50%
Captopril	[26]	25 mg q8 h	100%	75%	50%
Cefixime	[25, 27]	200 mg q12 h	100%	75%	50%
Cefotaxime	[26, 27]	1–2 g q6-12h	q8 h	q12 h	q12–24 h
Cephalexin	[24–26]	250–500 mg q6-8 h	q6 h or max 3g/d	q6-8 h or max 1.5g/d	q12 h or max 750mg/d
Cetirizine	[24]	5mg/d	100%	50%	Avoid
Chloroquine	[26]	310 mg/week	100%	100%	50%
Ciprofloxacin	[25, 27]	400 mg IV or 500–750 mg PO q12 h	q12 h or 100%	q12–24 h or 50–75%	q24 h or 50%
Diclofenac	[27]	25–75 mg q12 h	50%–100%	25%–50%	25%
Domperidone	[24]	10–20mg q6-8 h or 80mg/d	100%	Reduce dose	Reduce dose
Enalapril	[27]	5–10 mg q12 h	100%	75%	50%
Furosemide	[24]	40–120 mg PO/d	100%	100%	Need high doses
Gabapentin	[25, 27]	300–600 mg q8 h	100%	50%	25%
Levofloxacin	[27]	250–750 mg q24 h	100% q12 h	50% q12 h	50% q12 h
Linezolid	[24]	600mg q12 h	100%	100%	Reduce dose to avoid metabolite accumulation
Lisinopril	[25, 27]	5–10 mg/d	100%	50–75%	25–50%
Mefenamic Acid	[24]	500 mg q8 h	100%	100%	Avoid
Metoclopramide	[26, 27]	10–15 mg q8 h	100%	75%-100%	50%-75%
Ofloxacin	[27]	200–400 mg q12 h	q12 h	q12–24 h	q24 h
Paracetamol	[26, 27]	500-1000mg q8 h	q4 h	q6 h	q8 h
Piperacillin and Tazobactam	[27]	3.375–4.5g q6-8 h	q4–6 h	2.25g q6–8 h	2.25g q8 h
Pregabalin	[24]	75-300mg/d	100%	25-150mg/d	25-75mg/d
Ranitidine	[27]	150–300 mg at bedtime	100%	75%	25%
Rosuvastatin	[24]	5–40 mg/d	5-20mg/d	5mg/d or Avoid	Avoid
Sodium Bicarbonate	[24]	3g q2 h	100%	Caution	Caution or Avoid
Spironolactone	[25–27]	50–100 mg/d	100% or q6-12h	100% or q12-24 h	Avoid
Tranexamic Acid	[27]	25 mg/kg q6-8 h	50%	25%	10%
Vancomycin	[27]	1 g IV q12 h	q12 h	q24–36h	q48–72 h

At the end, the doses of drugs were assessed for appropriateness individually for each and every patient using these dose adjustment guidelines.

### Data Analysis

Statistical analysis was carried out using IBM SPSS version 20. Descriptive statistics were applied on independent variables such as the age, gender, stages of chronic kidney disease, the number of prescribed drugs, the length of hospitalization of patients, comorbidities of patients, and the pattern of medication dosing errors. Similarly, the descriptive statistics were applied to assess the central tendencies of dose adjustment numerical variables. The binary logistic regression analysis was done using following independent variables to assess the predictors of medication dosing errors in chronic kidney disease patients: Gender (Male, Female), Age (< 20 years, 20–40 years, 41–60 years, > 60 years), CKD Stage (Stage 3, Stage 4, Stage 5), Length of hospitalization (< 7 days, ≥ 7 days), Number of prescribed medicines (< 5, ≥ 5), Comorbidity (Yes, No), Diabetes (Yes, No), Hypertension (Yes, No), and Antibiotics prescribed (Yes, No). Variables with a p-value <0.1 in the univariate analysis were further studied in the multivariate analysis. A p-value <0.05 was considered significant throughout the statistical analysis.

## Results

In this study, nearly 205 medical charts of chronic kidney disease (CKD) patients were assessed. Of which, nearly 61.0% patients were males and 39.0% were females. Nearly 92 (44.9%) patients were aged between 20–40 years, followed by 60 (29.3%) who were aged between 41–60 years, and only 10.7% who had an age above 60 years. The mean age of CKD patients was 38.64 ±16.82 (SD) years. The average length hospitalization of CKD patients was 8.95 days (Range: 1–31 days). Furthermore, it was observed that the majority N = 155 (75.6%) of patients had CKD stage 5, followed by nearly 15.1% who had CKD stage 4, and the remaining 9.3% patients had CKD stage 3. The Hypertension (69.3%), Diabetes (19.1%) and Hepatitis C (4.8%) were observed as three topmost comorbidities in CKD patients (Table 2).

**Table 2:** Patient characteristics.

Variables		Frequency	%
**Age**	< 20	31	15.1
	20–40	92	44.9
	41–60	60	29.3
	> 60	22	10.7
**Gender**	Male	125	61.0
	Female	80	39.0
**Length of hospitalization**	< 7 days	97	47.3
	≥ 7 days	108	52.7
**Number of medicines**	< 5 drugs	45	22.0
	≥ 5 drugs	160	78.0
**CKD Stage**	Stage 3	19	9.3
	Stage 4	31	15.1
	Stage 5	155	75.6
**Comorbidity present**	Yes	159	77.6
	No	46	22.4
**Antibiotics prescribed**	Yes	156	76.1
	No	49	23.9
**Comorbidities**†	Hypertension	146	69.3
	Diabetes	40	19.5
	Hepatitis C	10	4.8
	Anemia	7	3.3
	Urinary Tract Infection	5	2.4
	Renal Stone	4	1.9
	System Lupus Erythematous	3	1.4
	Ischemic Heart Disease	3	1.4
	Hepatitis B	2	1.0

† = Variable with tick multiple option (Individual frequencies are based on total 205 patients)

Further analysis showed that overall 1534 drugs were prescribed to CKD patients, of which, nearly 522 (34.0%) required dosage adjustment. Of those 522 drugs, the majority N = 304 (58.2%), were unadjusted, and the remaining N = 218 (41.8%) were properly adjusted (Table 3).

**Table 3:** Frequency and central tendencies of adjusted and unadjusted prescribed drugs.

Variable	Frequency	Percentage	Mean ± SD	Median (IQR)
Total drugs prescribed	1534	100.0%	7.48 ± 2.551	-
Number of drugs requiring dose adjustment	522/1534	34.0%	-	2 (3)
Number of drugs properly adjusted	218/522	41.8%	-	1 (2)
Number of drugs unadjusted	304/522	58.2%	-	1 (1)

IQR (Interquartile range), SD (Standard deviation)

Moreover, the descriptive statistics revealed that the most common unadjusted drugs were amikacin (100.0%), linezolid (100.0%), cephalosporin antibiotics (100.0%), chloroquine (100.0%), spironolactone (100.0%), amoxicillin (79.4%), metoclopramide (87.5%), sodium bicarbonate (97.9%), rosuvastatin (71.4%), tranexamic acid (85.7%) ranitidine (65.5%), paracetamol (79.3%), domperidone (88%), and acetylsalicylic acid (81.8%). In contrast, the most accurately adjusted drugs were captopril (90.7%), bisprolol (66.6%), furosemide (52.6%), atenolol (58.8%), lisinopril (68.7%), and ofloxacin (100.0%), ciprofloxacin (91.3%), piperacillin/tazobactam (63.6%), vancomycin (66.6%), gabapentin (100%), pregabalin (100.0%) and cetirizine (100.0%). For further details please see Table 4.

**Table 4:** Pattern of medication dosing errors in chronic kidney disease patients.

ATC Classification	Drug Name	N of drugs needing adjustment	N (%) of adjusted drugs	N (%) of unadjusted drugs
**Alimentary tract and metabolism**	Metoclopramide	72	9 (12.5)	63 (87.5)
	Sodium Bicarbonate	48	1 (2.1)	47 (97.9)
	Ranitidine	32	11 (34.4)	21 (65.6)
	Domperidone	25	3 (12.0)	22 (88.0)
	Acetylsalicylic acid	11	2 (18.2)	9 (81.8)
**Antiinfectives for systemic use**	Ciprofloxacin	46	42 (91.3)	4 (8.7)
	Amoxicillin	34	7 (20.6)	27 (79.4)
	Piperacillin/Tazobactam	11	7 (63.6)	4 (37.4)
	Vancomycin	6	4 (66.6)	2 (33.3)
	Amikacin	4	-	4 (100.0)
	Levofloxacin	2	1 (50.0)	1 (50.0)
	Linezolid	2	-	2 (100.0)
	Cefixime	1	-	1 (100.0)
	Cefotaxime	1	-	1 (100.0)
	Cephalexin	1	-	1 (100.0)
	Ofloxacin	1	1 (100.0)	-
**Antineoplastic and immunomodulating agents**	Azathioprine	3	1 (33.3)	2 (66.6)
**Antiparasitic products, insecticides and repellents**	Chloroquine	1	-	1 (100.0)
**Blood and blood forming organs**	Tranexamic Acid	7	1 (14.3)	6 (85.7)
**Cardiovascular system**	Captopril	54	49 (90.7)	5 (9.3)
	Furosemide	38	20 (52.6)	18 (47.4)
	Atenolol	17	10 (58.8)	7 (41.2)
	Lisinopril	16	11 (68.7)	5 (31.3)
	Enalapril	12	8 (66.6)	4 (33.3)
	Amiloride	9	4 (44.5)	5 (55.5)
	Rosuvastatin	7	2 (28.6)	5 (71.4)
	Spironolactone	7	-	7 (100.0)
	Bisprolol	3	2 (66.6)	1 (33.3)
**Musculoskeletal system**	Diclofenac	6	2 (33.3)	4 (66.66)
	Mefenamic acid	2	-	2 (100.0)
**Nervous system**	Paracetamol	29	6 (20.7)	23 (79.3)
	Gabapentin	2	2 (100.0)	-
	Pregabalin	1	1 (100.0)	-
**Respiratory system**	Cetirizine	2	2 (100.0)	-
	Sum	513	209	304
	Others	9	9	-
**Total**		**522**	**218**	**304**

The logistic regression analysis was carried out to assess the predictors of medication dosing errors in CKD patients. The multivariate analysis confirmed that out of the nine studied variables, only the stages of chronic kidney disease i.e. CKD stage 4 (OR 0.054; 95% CI [0.017–0.177]; p <0.001) and CKD stage 5 (OR 0.098; 95% CI [0.040–0.241]; p <0.001), the number of prescribed medicines ≥ 5 (OR 0.306; 95% CI [0.133–0.704]; p 0.005), and the presence of a comorbidity (OR 0.455; 95% CI [0.226–0.916]; p 0.027) such as the hypertension (OR 0.453; 95% CI [0.231–0.887]; p 0.021) were associated with the medication dosing errors (Table 5).

**Table 5:** Predictors of medication dosing errors in chronic kidney disease patients.

Variables		N (patients)	patients with unadjusted drugs	UA (OR [95% CI])	p value	MA (OR [95% CI])	p value
**Gender**	Female	80	59 (73.8%)	Ref		Ref	
	Male	125	92 (73.6%)	1.062 [0.564–1.999]	0.852	-	-
**Age**	< 20	31	21 (67.7%)	Ref		Ref	
	20–40	92	66 (71.7%)	0.863 [0.261–2.853]	0.809	-	-
	41–60	60	48 (80.0%)	0.931 [0.329–2.631]	0.892	-	-
	> 60	22	16 (72.7%)	1.500 [0.484–4.651]	0.483	-	-
**CKD Stage**	Stage 3	19	5 (26.3%)	Ref		Ref	
	Stage 4	31	12 (38.7%)	0.056 [0.018–0.172]	**<0.001**	0.054 [0.017–0.177]	**<0.001**
	Stage 5	155	134 (86.5%)	0.099 [0.042–0.233]	**<0.001**	0.098 [0.040–0.241]	**<0.001**
**Length of hospitalization**	< 7 days	97	69 (71.1%)	Ref		Ref	
	≥ 7 days	108	82 (75.9%)	0.825 [0.445–1.528]	0.540	-	-
**Number of medicine**	< 5 drugs	45	25 (55.6%)	Ref		Ref	
	≥ 5 drugs	160	126 (78.8%)	0.355 [0.178–0.710]	**0.003**	0.306 [0.133–0.704]	**0.005**
**Comorbidity**	No	46	28 (60.9%)	Ref		Ref	
	Yes	159	123 (77.4%)	0.458 [0.231–0.909]	**0.025**	0.455 [0.226–0.916]	**0.027**
**Diabetes**	No	165	118 (71.5%)	Ref		Ref	
	Yes	40	33 (82.5%)	0.535 [0.220–1.288]	0.162	-	**-**
**Hypertension**	No	59	36 (61.0%)	Ref		Ref	
	Yes	146	115 (78.8%)	0.422 [0.219–0.814]	**0.010**	0.453 [0.231–0.887]	**0.021**
**Antibiotics prescribed**	No	49	34 (69.4%)	Ref		Ref	
	Yes	156	117 (75.0%)	0.756 [0.372–1.533]	0.437	-	**-**

UA (univariate analysis), MA (multivariate analysis); Significance: p < 0.05.

## Discussion

The present study showed that nearly 34.0% of drugs prescribed to chronic kidney disease (CKD) patients required dose adjustment. Of which, only 41.8% drugs were properly adjusted, and the remaining 58.2% were unadjusted. Amazingly, the medication dosing errors in the present study were much lower than the studies reported from Palestine, India and South Africa, whereby the percentages of unadjusted drugs were nearly 73.6%, 81.1%, and 59.0% respectively [9, 28, 29]. The comparatively lower percentage of unadjusted drugs in Pakistan could be due to differences in the selected patients in the abovementioned studies. The lower medication dosing errors as compared to other underdeveloped countries could also be due to the better knowledge of practicing physicians regarding the management of chronic kidney disease, as the CKD patients in the present study received medical care directly from trained nephrologists.

In marked contrast, the same percentage of medication dosing errors in Pakistan was comparatively higher than the studies reported from Bosnia and Herzegovina, Australia, France, Saudi Arabia, Indonesia, and even Nepal, whereby the percentages of unadjusted drugs were nearly 52.6%, 44.8%, 34.0%, 53.1%, 20.0%, and 13.5% respectively [7, 8, 12, 18, 30, 31]. This clearly reflects that Pakistani physicians are lacking knowledge as compared to those in the developed countries [32]. The better dose adjustment in CKD patients in high income countries could be due to the incorporation of advanced computerized dose adjustment systems and clinical pharmacists [33, 34]. Beside developed countries, the Nepal and Indonesia have also incorporated clinical pharmacists in their clinical settings that perhaps resulted in lower medication dosing errors. Therefore, we are suspecting that the lack of clinical pharmacists and computerized dose adjustment programs in the Pakistani clinical settings could have caused higher medication dosing errors. In addition, several other factors such as the negligence and traditional clinical practices of physicians, the lack of laboratory support, and the lack of standard dosing guidelines could also have caused higher medication dosing errors in our study setting [8, 9].

Another important aspect of the present study is the strange age distribution of CKD patients, which is likely different from those in the developed and the underdeveloped countries [8, 12, 30]. This difference could be due to several reasons. For instance, the average life expectancy of a normal person in Pakistan is only 65 years, which is much lower than the western counterparts [35]. Therefore, there are likely chances that the CKD patients are dying at an earlier age, even before reaching the national average life-expectancy. There is another possibility that the prevalence of CKD is itself higher in the younger population due to a higher prevalence of hypertension, diabetes and smoking in Pakistan [36, 37]. A recent study has also reported that the prevalence of CKD is higher in patients aged <50 years in Pakistan, and the underlying causes of CKD were the glomerulonephritis, diabetic nephropathy, renal stones and the hypertension [38]. However, further studies are required to assess both these aspects as reliable evidence is lacking.

Furthermore, the assessment of the pattern of medication dosing errors has revealed that the majority of drugs prescribed without any dose adjustment were metoclopramide, sodium bicarbonate, ranitidine, domperidone, acetylsalicylic acid, amoxicillin, linezolid, cephalosporin antibiotics, diclofenac, mefenamic acid, spironolactone, and rosuvastatin. These findings are in line with previous studies with the exception of ciprofloxacin and the cardiovascular medicines that were prescribed more appropriately in our study setting [9, 21]. These findings show that physicians working in the Pakistani public sector hospitals are underestimating the adverse outcomes associated with several important medicines. For instance, the medicines such as amikacin, and cephalosporin antibiotics that are well reported to induce nephrotoxicity [21]. Moreover, the linezolid use has also been linked with the CKD and thrombocytopenia, therefore, the dose of linezolid should also be adjusted in order to reduce the likelihood of thrombocytopenia in CKD patients [39]. However, the Pakistani nephrologists should be commended for adjusting several important medicines such as the cardiovascular medicines, piperacillin/tazobactam, ofloxacin, ciprofloxacin, vancomycin, gabapentin, and pregabalin.

Lastly, the regression analysis confirmed that the age, gender, and the lengthy hospitalization of patients were not associated with the medication dosing errors. These findings are in line with previous studies elsewhere [9, 21]. However, the number of prescribed medicines ≥ 5, the presence of a comorbidity, such as hypertension, and the severe-to-end stages of chronic kidney disease were significantly associated with the medication dosing errors. To the best of our knowledge, these findings are comparatively new and not studied before in the developing countries. We also found that diabetes, that is one of the major underlying causes of CKD, was not associated with medication dosing errors, which is in contrast to an Australian study [20]. Moreover, the antibiotic prescribing was also not associated with medication dosing errors. Therefore, attention should be paid to the abovementioned significant predictors of medication dosing errors and thus the doses of drugs should be prescribed carefully and appropriately to avoid the risk of drug related toxicities and adverse outcomes.

### Strengths and Limitations

There present study has several strengths. For instance, this is the first study of its kind that is performed in Pakistan. Second, as compared to the past more detailed pattern and predictors of medication dosing errors were studied and identified. Despite these strengths, the study has several limitations. First, a retrospective study design was employed which restricted us from suggesting interventions and observing actual adverse drug reactions. Second, the MDRD equation was used due to lack of data regarding patients’ weight, which is considered unsuitable for patients with higher muscle mass and for those suffering from serious disorders such as cancer [40]. Third, due to the lack of data, the most responsible diagnosis for hospital admission could not be identified, and the diagnosis of CKD could not be verified using the baseline serum creatinine values. Moreover, the lack of data also restricted us to study only those charts that had mentioned serum creatinine values. Fifth, due to the lack of funding, the present study was conducted at only one tertiary care hospital, therefore, these findings should be carefully generalized to across the Pakistan.

### Clinical Implications and Future Recommendations

The medication dosing errors in CKD patients is a global concern. It has been reported that CKD alters the pharmacokinetic parameters of drugs such as the bioavailability, protein binding, biotransformation, volume of distribution, and renal excretion of drugs which makes CKD patients more prone to severe adverse outcomes [9]. Therefore, physicians should take extra care while prescribing drugs to CKD patients.

One important point that we want to mention here is; due to the lack of consensus among dose adjustment guidelines, confusion exists among nephrologists regarding the dose adjustment of several renally excreted drugs. The best example here is sodium bicarbonate. Some studies and guidelines show that it is beneficial for CKD patients and some do not [24, 41]. Therefore, it is suggested that nephrologists should make a universal consensus and develop internationally applicable dose adjustment guidelines to ensure appropriate drug dose adjustment to save CKD patients from drug related adverse outcomes.

Furthermore, the clinical pharmacists should be incorporated in clinical settings, especially in the developing countries, and allowed to intervene and ensure safe prescribing in CKD patients by playing their role as an active healthcare team member. Additionally, educational programs should be designed to improve and refresh the knowledge of physicians regarding the management of CKD. Apart from that, further research should be conducted to assess the impact of more variables such as the specific drug classes and the specific comorbidities on the medication dosing errors in chronic kidney disease patients. In addition, epidemiological studies should be done to assess the changing pattern of CKD disease and to identify the underlying causes of CKD disease especially in the developing countries such as Pakistan.

## Conclusions

It is concluded that the occurrence of medication dosing errors was high in hospitalized chronic kidney disease patients in Pakistan. More than half drugs prescribed to CKD patients requiring dose adjustment, were unadjusted. The predictors of medication dosing errors in CKD patients were the severe-to-end stages of chronic kidney disease, the number of prescribed medicines ≥ 5, and the presence of a comorbidity such as hypertension. Therefore, physicians are advised to take care of these predictors of medication dosing errors while prescribing drugs to chronic kidney disease patients to minimize the risk of drug related toxicities and adverse outcomes.

## Supporting Information

S1 Table Data Collection Form. (PDF)
