# Supplementary material for: Pattern and Predictors of Medication Dosing Errors in Chronic Kidney Disease Patients in Pakistan: A Single Center Retrospective Analysis
Source: PLoS One. 2016 Jul 1;11(7):e0158677. doi: 10.1371/journal.pone.0158677 (PMC4930166; doi:10.1371/journal.pone.0158677)
Supplement: S1 Table — (PDF) [file pone.0158677.s001.pdf]

DATA COLLECTION FORM

Patient Number (Ref): ______________________ Date of Admission: ________________ Date of Discharge: _______________ Length of Stay (days): _________

Gender: __________________________________ Age of Patient: ____________________ Weight: _______________ Serum Creatinine: ___________________

Underlying Disease: ____ AND/OR Co-morbidity: ____

CrCl (MDRD): ____

Total Number of Medicines: _________________ Total Number of Drugs requiring adjustment: _________________ No of adjusted drugs: ________________

TREATMENT PRECRIBED									
**Drug Prescribed**	**Generic Name**	**Qty.**	**Route**	**Dose**	**Frequency**	**Daily Dose**	**Estimated dose**	**Adjustment (1/0)***	**Adjusted (1/0)****
1.									
2.									
3.									
4.									
5.									
6.									
7.									
8.									
9.									
10.									
11.									

* 1=Required, 0=Not Required; ** 1=Yes, 0=No
